# Diagnosis and treatment of late-onset Pompe disease in the Middle East and North Africa region: consensus recommendations from an expert group

**DOI:** 10.1186/s12883-015-0412-3

**Published:** 2015-10-15

**Authors:** The MENA Pompe Working Group, Fatma Al Jasmi, Mohammed Al Jumah, Fatimah Alqarni, Nouriya Al-Sanna’a, Fawziah Al-Sharif, Saeed Bohlega, Edward J. Cupler, Waseem Fathalla, Mohamed A. Hamdan, Nawal Makhseed, Shahriar Nafissi, Yalda Nilipour, Laila Selim, Nuri Shembesh, Rawda Sunbul, Seyed Hassan Tonekaboni

**Affiliations:** Department of Pediatrics, College of Medicine and Health Science, United Arab Emirates University, P.O. Box 17666, Al-Ain, United Arab Emirates; King Abdullah International Medical Research Center, King Saud Bin Abdulaziz University for Health Sciences, NGHA, Riyadh, Kingdom of Saudi Arabia; Prince Mohammed Ben Abdulaziz Hospital, MOH, P.O. Box 22490, Riyadh, 11426 Kingdom of Saudi Arabia; Neurology Department, National Neurosciences Institute, King Fahad Medical City, P.O. Box 59046, Riyadh, 11525 Kingdom of Saudi Arabia; Johns Hopkins Aramco Healthcare, Pediatrics Services Division, Building 61/Room D-269, Dhahran, Kingdom of Saudi Arabia; Medical Genetics And Metabolic Consultant, MCH, PO Box 55954, Jeddah, 21544 Kingdom of Saudi Arabia; Department of Neurosciences, MBC 76, King Faisal Specialist Hospital and Research Centre, P.O. Box 3354, Riyadh, 11211 Kingdom of Saudi Arabia; Department of Neuroscience, MBC J-76, King Faisal Specialist Hospital and Research Center, P.O. Box 40047, Jeddah, 21499 Kingdom of Saudi Arabia; Department of Pediatrics, Division of Child Neurology, Mafraq Hospital, P.O. Box: 2951, Abu Dhabi, United Arab Emirates; KidsHeart: American Fetal & Children’s Heart Center, Dubai Healthcare City, P.O. Box 505193, Dubai, United Arab Emirates; Pediatric Department, Jahra Hospital, Ministry of Health, P.O. Box 16586, Qadisiya, 35856 Kuwait; Department of Neurology, Tehran University of Medical Sciences, Shariati Hospital, North Karegar Street, Tehran, 14114 Iran; Pediatric Pathology Research Center, Mofid Children Hospital, Shahid Beheshti Medical University (SBMU), Shariati Avenue, Tehran, 15468-155514 Iran; Pediatric Neurology and Neurometabolic Division, Cairo University Children Hospital (Abo el Reesh), 1-Aly Pasha Ibrahim Street, Near Sayeda Zeinab Metro Station, Cairo, Egypt; Pediatrics and Pediatric Neurology, Benghazi University, P.O. Box 1565, Benghazi, Libya; Department of Pediatrics, Qatif Central Hospital, P.O. Box 18476, Dammam, 31911 Eastern Province Kingdom of Saudi Arabia; Pediatric Neurology Research Center, Mofid Children Hospital, Shahid Beheshti Medical University (SBMU), Shariati Avenue, Tehran, 15468-155514 Iran

## Abstract

**Background:**

Pompe disease is a rare autosomal recessive disorder caused by a deficiency of the lysosomal enzyme alpha-glucosidase responsible for degrading glycogen. Late-onset Pompe disease has a complex multisystem phenotype characterized by a range of symptoms.

**Methods:**

An expert panel from the Middle East and North Africa (MENA) region met to create consensus-based guidelines for the diagnosis and treatment of late-onset Pompe disease for the MENA region, where the relative prevalence of Pompe disease is thought to be high but there is a lack of awareness and diagnostic facilities.

**Results:**

These guidelines set out practical recommendations and include algorithms for the diagnosis and treatment of late-onset Pompe disease. They detail the ideal diagnostic workup, indicate the patients in whom enzyme replacement therapy should be initiated, and provide guidance on appropriate patient monitoring.

**Conclusions:**

These guidelines will serve to increase awareness of the condition, optimize patient diagnosis and treatment, reduce disease burden, and improve patient outcomes.

## Introduction

Pompe disease, also known as acid maltase deficiency (AMD) or glycogen storage disease type II (GSDII), is a rare autosomal recessive disorder of glycogen metabolism caused by insufficient activity of the enzyme acid alpha-glucosidase (GAA). There are two forms of the disease: infantile-onset Pompe disease (IOPD; aged <1 year with cardiomyopathy) and late-onset Pompe disease (LOPD; >1 year of age through to adulthood or <1 year without cardiomyopathy). The worldwide incidence of both forms of the disease is commonly reported to be 1 in 40,000 [1], however prospective trials suggest the incidence may be as high as approximately 1 in 9,000 [2, 3].

Myozyme® (alglucosidase alfa) was approved for the treatment of Pompe disease in Europe and the United States in 2006 and has changed the management of Pompe disease, improving the symptoms of the disease in many, but not all, patients [4]. Additionally, enzyme replacement therapy (ERT) with alglucosidase alfa extends life expectancy in patients with LOPD [5].

A number of guidelines exist for the diagnosis and treatment of LOPD [6–8]; however these do not address the challenges faced by the Middle East and North Africa (MENA) region in the diagnosis and treatment of LOPD, namely delayed diagnosis, access to diagnostic tools, and access to treatment. There is, therefore, a need for region-specific recommendations for the diagnosis and treatment of LOPD which assess the latest available literature and tailor it to the region with due consideration of local experiences and challenges. The objective of this paper is to provide clinical guidelines on the diagnosis and treatment of LOPD in the MENA region.

## Methods

A panel of experts met to develop consensus recommendations to aid diagnosis and treatment of infantile- and late-onset Pompe disease in the MENA region. The panel of physicians from across the MENA region included experts from different specialties – adult and pediatric neurology, metabolic diseases, genetics, pediatric cardiology, and neuropathology – all with expertise in the diagnosis and/or management of Pompe disease.

A literature review was performed prior to the meeting. A search for relevant articles including randomized control trials, review articles, and most recent international guidelines on the diagnosis and treatment of LOPD was undertaken using PubMed. The search terms included: *late onset Pompe disease*, *glycogen storage disease type II*, *lysosomal storage disorders*, *acid alpha-glucosidase deficiency*, and *acid maltase deficiency*.

The panel critically analyzed and discussed these articles at the meeting. The validity, clinical relevance, and applicability of the evidence for LOPD in the MENA region were discussed. After considering the evidence, the panel achieved a consensus on a number of recommendations that are supported by best scientific evidence.

## Background

Pompe disease was first characterized in 1932 by Joannes C. Pompe, who described glycogen accumulation in cardiac muscle in a 7-month-old girl who died [9]. Pompe disease was first categorized as GSDII by G.T. Cori in 1954 [10], and further understanding of the disease was achieved with the discovery of lysosomes in 1955 by Christian de Duve [11, 12]. In 1963, Henri Hers identified GAA as the enzyme that is deficient in Pompe patients and therefore responsible for Pompe disease [13].

Following the discovery of GAA deficiency as the cause of Pompe disease, a number of enzyme replacement trials have been conducted. In 1964, Baudhuin and colleagues intramuscularly injected an extract of the fungus *Aspergillus niger*, which was shown to contain GAA, into a patient with abnormal lysosomes; however the treatment did not demonstrate any appreciable change in the lysosomes [14].

In 1974, ERT using enzymes purified from a human source were administered to two patients with Gaucher’s disease [15]. Exogenous glucocerebrosidase was found to cause a definite decrease in the quantity of accumulated lipids, but the results were largely disappointing. The first successful ERT of a lysosomal storage disorder in humans was achieved in 1991 when Barton and colleagues reported the results of infusion of macrophage-targeted human placental glucocerebrosidase into 12 patients with type 1 Gaucher’s disease [16]. In the same year, the first successful *in vivo* study of GAA ERT was reported [17]. Van der Ploeg and colleagues reported that a human placenta-derived GAA delivered by a mannose 6-phosphate receptor-mediated ERT had resulted in cardiac uptake in mice, offering the potential for a treatment for Pompe disease.

This potential was realized in 2001 when the first clinical studies of ERT infusion in IOPD patients were published [18, 19]. These showed that recombinant human GAA (rhGAA) derived from either Chinese hamster ovary (CHO) cells or transgenic rabbit milk is capable of improving cardiac and skeletal muscle function. Longer term studies confirmed the tolerability, safety, and efficacy of ERT in Pompe disease [20, 21], resulting in the regulatory approvals of alglucosidase alfa for the treatment of Pompe disease in 2006 [22].

The first randomized controlled study of ERT in LOPD patients was published in 2010 [23] and demonstrated that alglucosidase alfa leads to stabilization of neuromuscular deficits as well as some degree of functional improvement [23, 24].

ERT with alglucosidase alfa is not a cure for Pompe disease, but it can greatly modify or attenuate the phenotype, improving clinical outcome, quality of life (QOL), and survival.

## Epidemiology

The combined incidence of both forms of Pompe disease varies depending on ethnicity and geographic region, ranging from 1 in 14,000 to 1 in 600,000 reported in African American and Portuguese populations, respectively [25–27].

The worldwide incidence of both forms of the disease is commonly reported to be 1 in 40,000 [1]. The frequency of LOPD is estimated at 1 in 57,000 [28]. However, two prospective trials performed in the context of newborn screening challenge this. The incidence of LOPD in Taiwan based on a prospective newborn screening study is 1/26,466 [2] and the overall incidence of Pompe disease in Austria based on a prospective newborn screening study is 1/8,684 live births [3].

There are no late-onset incidence studies in the MENA region; however the prevalence of IOPD in Emiratis has been reported as 2.66 per 100,000 based on UAE experience from two centers and is likely much higher [29]. Additionally, the high degree of consanguinity in the region results in higher incidence of autosomal recessive diseases in general [30].

## Pathophysiology

**Fig. 1:**
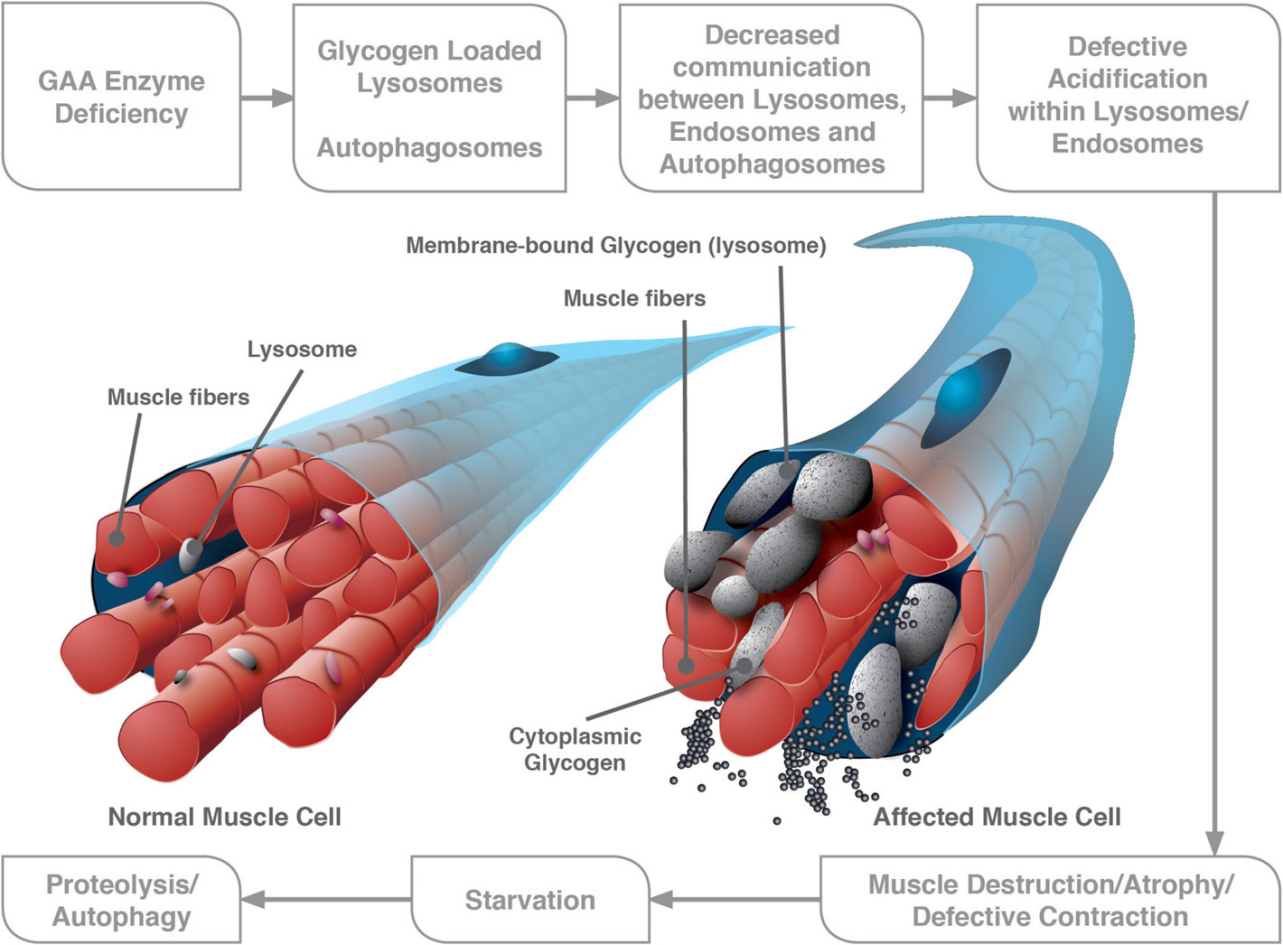
Pathophysiology of late-onset Pompe disease. Abbreviations: GAA, acid alpha-glucosidase

Pompe disease is a result of mutations in the *GAA* gene, which is located on the long arm of chromosome 17 (17q25.2-q25.3) [31, 32] and encodes the 105-kDa GAA enzyme [33]. Mutations in the gene lead to deficiency in the lysosomal enzyme GAA, causing accumulation of lysosomal and non-lysosomal glycogen in multiple tissues [34]. Lysosomal enlargement and rupture, as well as autophagic accumulation, contribute to the progressive myopathy in the disease, with autophagic accumulation causing skeletal muscle destruction in LOPD (Fig. 1).

Due to the broad clinical spectrum of the presenting phenotype, a number of differential diagnoses for Pompe disease should be considered. These differential diagnoses and their features common to Pompe disease are summarized in Table 1.

**Table 1:** Differential diagnosis of LOPD

Differential condition	Common symptoms
Limb–girdle muscular dystrophy (LGMD)	Progressive muscle weakness in the pelvis, legs, and shoulders; elevated CK
Becker muscular dystrophy (BMD)	Progressive proximal muscle weakness, prominent quadriceps weakness, calf hypertrophy, elevated CK, cardiomyopathy
Selenoprotein N1-related myopathy	Spinal rigidity, respiratory failure, muscle hypotrophy
Myasthenia gravis	Ptosis, ophthalmoplegia, bulbar dysfunction, proximal muscle weakness, fluctuating course
Spinal muscular atrophy	Progressive proximal muscle weakness and atrophy, respiratory failure, postural tremor, mild elevated CK
Polymyositis	Unexplained muscle weakness with elevated CK
Glycogen storage diseases: IIIa (Debrancher deficiency/Cori), IV (branching enzyme deficiency/Anderson disease), V	Hypotonia, hepatomegaly and hepatic failure, muscle weakness with distal involvement, elevated CK
Danon disease	Hypertrophic cardiomyopathy, skeletal muscle myopathy
Mitochondrial myopathies	Hyptonia, hyporeflexia, hepatomegaly. Some forms with hypertrophic cardiomyopathy, muscle weakness, external ophthalmoplegia, elevated CK
Lipid storage myopathies	Fluctuating muscle weakness with respiratory involvement, sometimes bulbar weakness, elevated CK

*CK* creatine kinase. Adapted by permission from Macmillan Publishers Ltd: Genet Med. 2006;8(5):267–88 [52] © 2006

## Clinical phenotype

LOPD has a wide spectrum of signs and symptoms [1]. While the age of symptom onset has been reported from <12 months of age to 70+ years, the average age at onset of symptoms is between 29 and 33 years, while the mean age at initial diagnosis is 36–43 years [35–38].

LOPD is a multisystem disorder which typically manifests as limb–girdle muscle weakness, respiratory symptoms, and progression to respiratory insufficiency due to diaphragmatic and intercostal muscle weakness. The phenotype can include varying degrees: limb–girdle muscle weakness, scapular winging, neck flexor weakness, abdominal wall musculature involvement, or a lordotic waddling gait [1]. Symptoms may include rigid spine syndrome (RSS), scoliosis, low body mass [38], and asymptomatic hyperCKemia [39]. Other symptoms may include asymmetrical ptosis, tongue weakness, and dysphagia [35, 38, 40, 41]. Myalgia and fatigue are important symptoms as they are sometimes the presenting complaint [35].

Cardiac symptoms are common in IOPD and while cardiac involvement in LOPD remains somewhat controversial with conflicting reports found in the literature, Wolff–Parkinson–White syndrome has been implicated. A study of 46 consecutive adult patients with LOPD suggests that major cardiac abnormalities are rare [42]. However, another study of the clinical and neurophysiological spectrum of 38 LOPD patients identified three subjects who suffered from arrhythmias due to Wolff–Parkinson–White syndrome despite no echocardiographic signs of cardiomyopathy [1]. Cerebral aneurysms have been reported as complications in LOPD patients, and may be increased in incidence over the general population, however this is not certain [43, 44].

Hearing loss is often described in classic IOPD patients. While it is rarer in LOPD, there have been reports of mild hearing loss due to stapedius muscle or cochlear involvement [45, 46].

Low bone mineral density, scoliosis, and osteoporosis have also been reported in LOPD patients. A study of 46 patients found that bone mineral density was significantly lower in Pompe patients than in healthy individuals, with 26 % of patients classified as having osteoporosis/low bone mass for chronological age; however this may be a consequence of weakness and reduced mobility, as seen in muscular disorders, and not specific for LOPD [47].

Recent studies have identified LOPD in both symptomatic patients with muscle weakness and patients presenting with presymptomatic hyperCKemia [48–50].

## Diagnosis

With such a wide variation in age of onset and a non-specific symptoms complex, a high degree of clinical suspicion is necessary to diagnose patients with LOPD and a low threshold should be used for clinical screening with dried blood spot (DBS) testing. The diagnosis should be suspected on the clinical presentation and phenotype and the evaluation should then ensue.

### Clinical evaluation

#### Creatine kinase

Serum creatine kinase (CK) is elevated in 95 % of patients with LOPD [51], however the range can vary from normal (60–305 IU/L) to 15 times the upper limit of normal. Concurrent elevation of alanine transaminase (ALT), aspartate transaminase (AST), and lactate dehydrogenase (LDH) from muscle sources are frequently detected [52]. Serum CK may be elevated in presymtomatic LOPD patients [48, 49].

#### Forced vital capacity

The forced vital capacity (FVC) should be performed in the upright and supine positions and can be done with a hand-held spirometer. In most normal subjects, FVC in the supine position is 5–10 % less than when upright; a drop of ≥10 % is suggestive of diaphragmatic weakness and a drop >30 % is associated with severe diaphragmatic weakness [53, 54].

#### Electromyography

A conventional electromyograph (EMG) may be normal in patients with LOPD, however studies suggest that approximately 70 % may have a myopathic EMG pattern [1]. Studying the more proximal muscles, including the paraspinal muscles, has been shown to be more likely to reveal abnormalities. In addition to typical myopathic features, myotonic discharges without clinical myotonia have also been described sometimes occurring only in paraspinal muscles [55]. In addition, fibrillation potentials, positive sharp waves, and complex repetitive discharges may be seen [41]. Nerve conduction studies in Pompe disease patients are typically normal [1].

#### Dried blood spot testing

When available, DBS testing for GAA activity is the preferred screening test for LOPD as it is a simple, inexpensive, and noninvasive assay [56]. Briefly, a blood spot obtained from the patient is assayed for GAA activity using spectrophoto-fluorimetry with recombinant GAA used as a calibrator for immuno-quantification [57]. The limitations of the DBS test include logistics, a lack of local facilities to perform the analysis, long turnaround times for testing in some regions of the world, and false-positive results.

A positive DBS is not diagnostic for LOPD and a second test should be performed to confirm the diagnosis. These confirmatory tests can include lymphocytes for GAA analysis, fibroblast cultures again assayed for GAA, gene sequencing, or muscle biopsy with or without GAA enzyme analysis. The diagnostic tests for LOPD are summarized in Table 2.

**Table 2:** Summary of diagnostic tests for LOPD

Test	Pompe presentation
Creatine kinase	Varies from normal to 15 times the upper limit of normal
Alanine transaminase and aspartate transaminase	Frequently elevated
Forced vital capacity	Reduced in most patients. A drop of ≥10 % in supine versus upright is suggestive of diaphragmatic weakness and a drop >30 % is associated with severe diaphragmatic weakness
Electromyography	Myopathic EMG may be present, particularly in proximal muscles such as the paraspinal muscles. Myotonic discharges without clinical myotonia, fibrillation potentials, positive sharp waves, and complex repetitive discharges may also be seen
Dried blood spot	GAA activity reduced

*EMG* electromyography, *GAA* acid alpha-glucosidase

#### Confirmatory tests

##### Lymphocytes for GAA

GAA activity cannot be assayed in mixed leukocytes as neutrophils contain maltase gluco-amylase (MGA) which leads to false-negative results [56]. Lymphocytes do not contain MGA and therefore LOPD can be diagnosed by assaying GAA in purified lymphocyte preparations. Testing lymphocytes for GAA is minimally invasive and has a rapid turnaround time. If successfully performed, a positive result may preclude the need for a more invasive test such as skin or muscle biopsy. The major limitation is that isolation and purification must be performed within 30 minutes and there is a lack of facilities and appropriately trained staff in most centers. Inclusion of acarbose is recommended to eliminate the interference by MGA when neutrophils are present [56].

##### Fibroblast cultures

Enzyme analysis of cultured fibroblasts is the gold standard test for diagnosis. A skin biopsy typically requires local anesthetic and requires 4–6 weeks of cell culture, after which an assay is used to quantify GAA activity. In children and adults with Pompe disease, residual GAA activity is approximately 2–40 % of normal activity [52]. A key advantage is that it may preclude the need for an invasive muscle biopsy; however, the main limitations are the possible failure of fibroblast growth, the long turnaround time (usually 6–8 weeks) and the need for cell culture and enzyme assay facilities.

##### Genetic testing

*GAA* is the only gene in which mutations are known to cause LOPD. Numerous pathogenic variants of the gene have been reported [58]. Mutations are spread across the gene, with particular mutations more common in some populations due to founder effects [52]. Methods for detecting mutations include targeted mutational analysis, full sequence analysis, and deletion/duplication analysis; however none of these have a 100 % mutation detection frequency [25]. The limitations of genetic testing include time, cost, and specificity.

##### Muscle biopsy

**Fig. 2:**
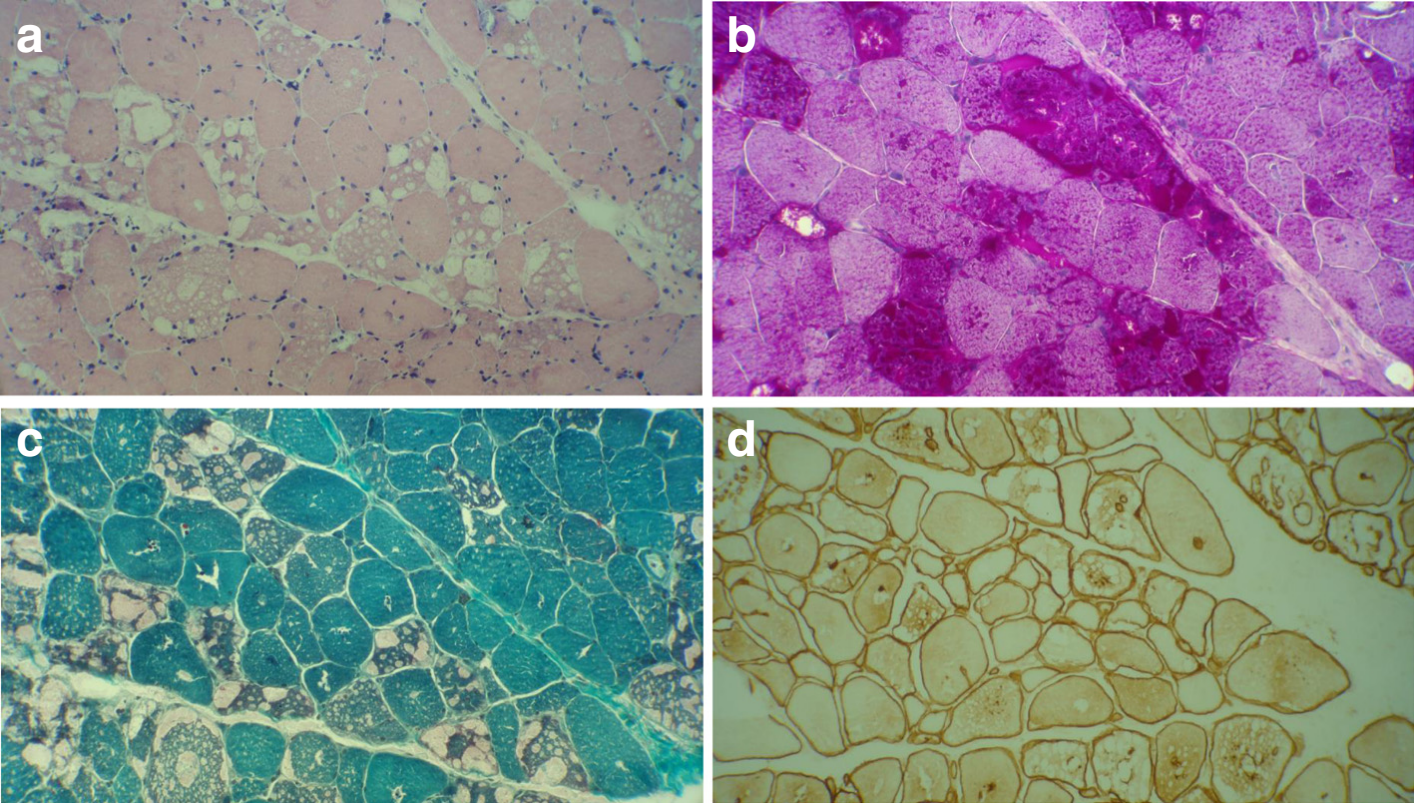
Biopsy from right vastus lateralis of a 46-year-old woman with LOPD. She had a long history of muscle weakness presenting with respiratory insufficiency. **a** Prominent fiber size variation and excess internalized nuclei with variable-sized subsarcolemmal and cytoplasmic vacuoles (H&E stain x200). **b** Pronounced vacuolation of many fibers, some vacuoles are slightly red-rimmed (modified Gomori trichrome stain x200). **c** Pronounced glycogen accumulation as intense staining of subsarcolemmal and cytoplasmic vacuoles (PAS stain x200). **d** Immunolabeling of multiple membrane-bound cytoplasmic vacuoles with dystrophin (Dystrophin 1, Biogenex Co. x200). Images provided by Dr Yalda Nilipour

**Fig. 3:**
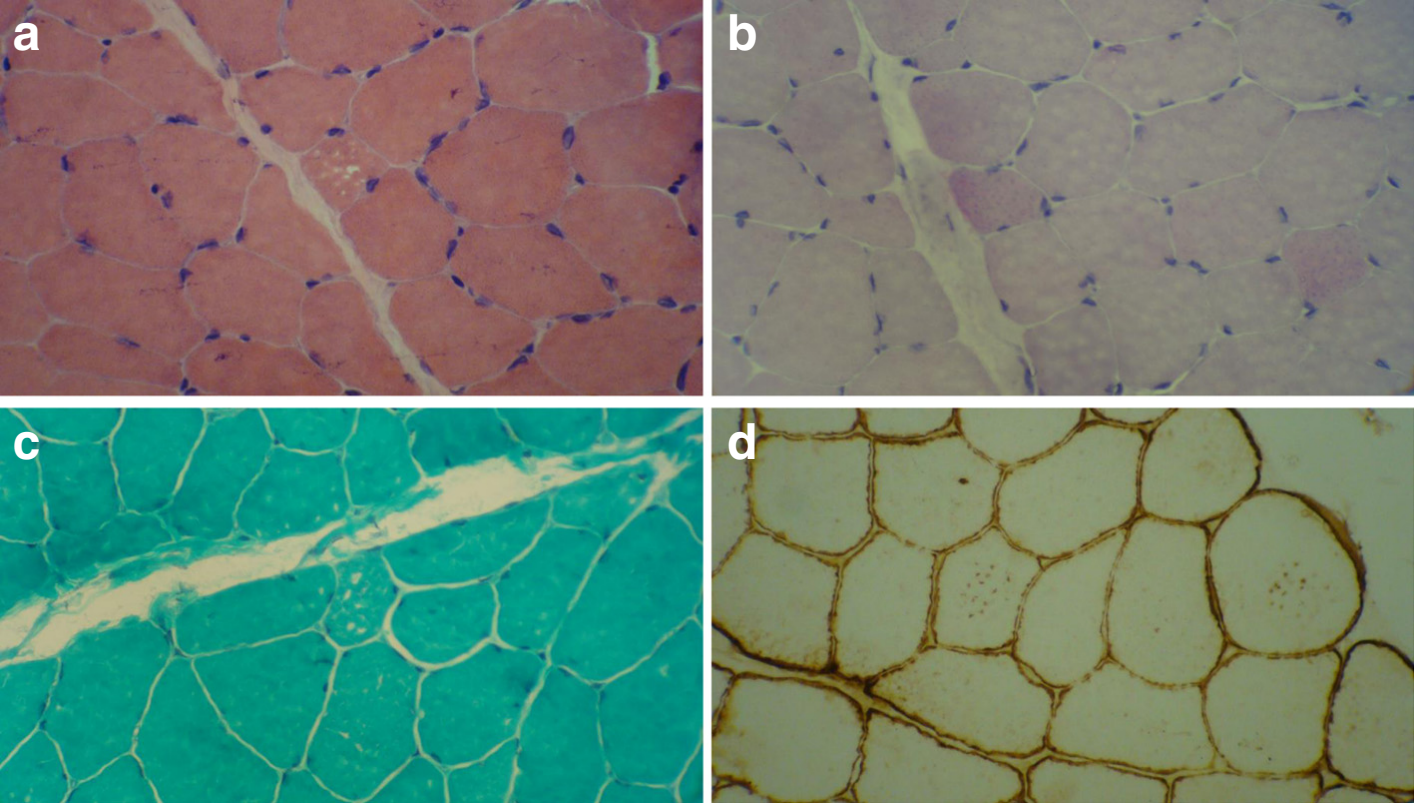
Muscle biopsy from left brachioradialis of a 40-year-old man with LOPD. He had had progressive limb–girdle muscle weakness since adulthood. **a** Slight variation in fiber size and some internalized nuclei with tiny cytoplasmic vacuoles in few fibers (H&E x400). **b** Only a little accumulation of glycogen in few fibers as punctate staining (PAS x400). **c** Tiny cytoplasmic vacuoles in few fibers. (Gomori trichrome x400). **d** Immunolabeling of dystrophin. Dot-like labelling of tiny membrane-bound cytoplasmic vacuoles in some fibers (Dystrophin 1, Biogenex Co. x400). Images provided by Dr Yalda Nilipour

Muscle biopsy is widely practiced in the assessment of patients with neuromuscular disorders. It can be used as a diagnostic or a confirmatory test in LOPD (Figs. 2 and 3). The procedure is invasive but most adult patients only require local anesthesia. The muscle specimen must be immediately frozen in isopentane, cooled in liquid nitrogen, and shipped on dry ice. Muscle biopsy in LOPD shows the presence of vacuoles that stain positively for glycogen, with accumulation in the lysosomes and cytosol observed in the advanced stages of the disease [52]. The limitations of muscle biopsy include the potential for false-negative results, the scarcity of facilities and personnel to process and interpret the specimens, and the invasive nature of the procedure.

In some cases, muscle biopsy can be normal or show non-specific changes using routine techniques of evaluation. Non-specific changes include lobulated or globular fibers, moth-eaten fibers, degenerative fibers, or even necrotic fibers and ragged-red-fibers or COX-negative fibers [59].

Up to 20–30 % of individuals with LOPD with documented decreased enzyme activity may not show muscle-specific changes and therefore a non-diagnostic muscle biopsy of adult patients does not exclude Pompe disease [60]. Biopsies from patients with LOPD have been read as normal, polymyositis, and others.

##### Whole body/muscle magnetic resonance imaging

Where it is available and feasible, whole body magnetic resonance imaging (MRI) can be used to better define the degree and distribution of muscle involvement and aid in the selection of muscles for biopsy [61]. Furthermore, early recognition of respiratory muscle involvement using MRI could allow an early start of ERT [62]. Although whole body MRI is now being included in clinical trials to monitor patients, its use in clinical practice is not yet recommended for monitoring individual patients.

### Diagnostic dilemmas

It has previously been reported that diagnosis of Pompe disease is a diagnostic dilemma in itself due to the rarity of the disease and the non-specific phenotype [52]. A number of diagnostic dilemmas are faced in the region. Mutation-related dilemmas include the identification of new variants with an unknown significance for pathology which requires the use of clinical findings and other biochemical parameters for diagnosis. Other diagnostic dilemmas include a positive screening test and only one mutation by genetic testing and positive DBS with unusual clinical features. In these cases the gold standard of fibroblast culture is recommended.

### Consensus recommendations

**Fig. 4:**
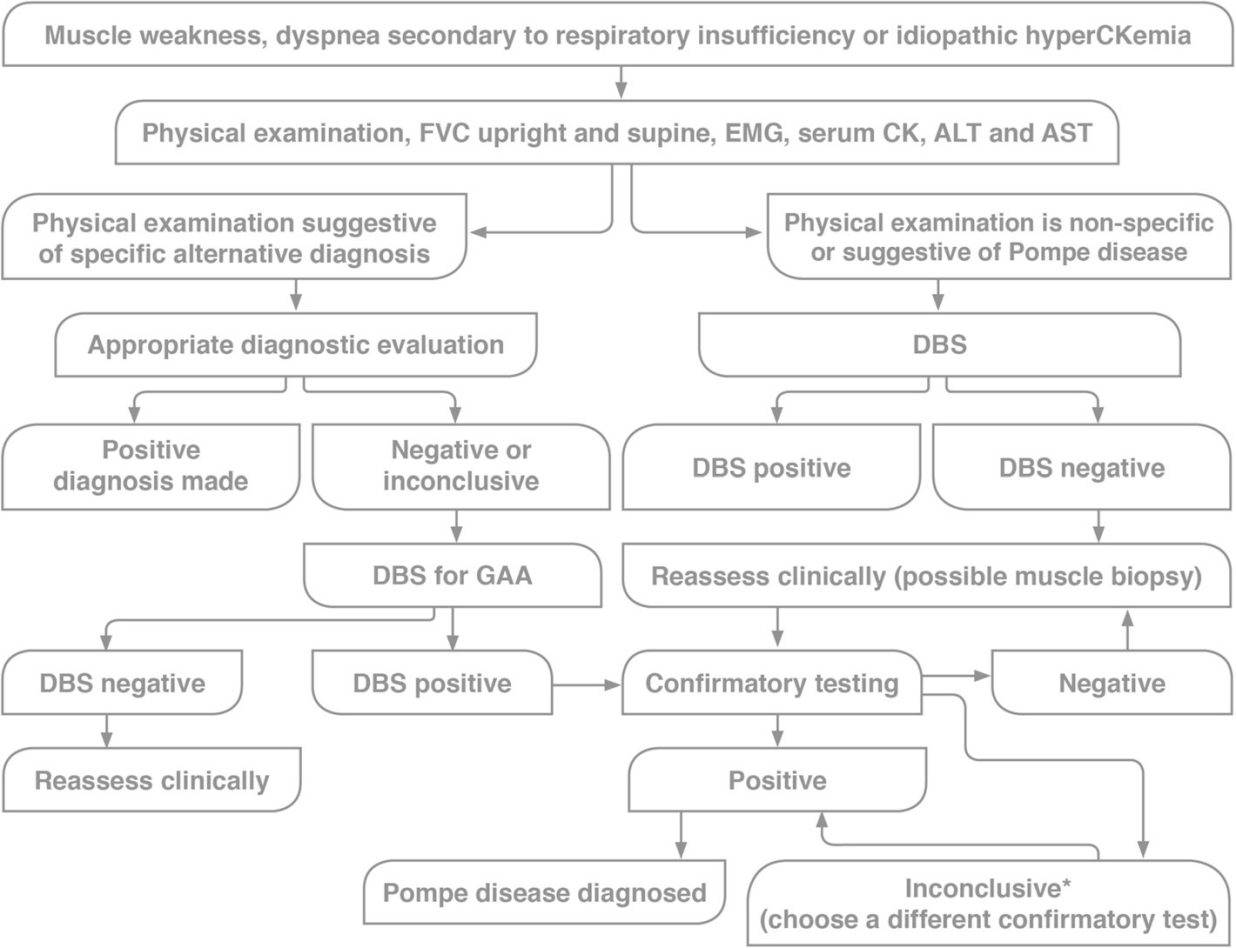
Algorithm for the diagnosis of LOPD. Abbreviations: ALT, alanine transaminase; AST, aspartate transaminase; CK, creatine kinase; DBS, dried blood spot; EMG, electromyography; FVC, forced vital capacity; GAA, acid alpha-glucosidase. *Alternative diagnosis made. Adapted with permission from AANEM. Diagnostic Criteria for Late-Onset (Childhood and Adult) Pompe Disease. Muscle Nerve. 2009;40:149–160 [107] © 2009 Wiley Periodicals, Inc

The experts agreed that the diagnosis of LOPD can be difficult, as the phenotype is heterogeneous and may resemble the clinical features of a number of other neuromuscular disorders; a high clinical suspicion was considered important for the diagnosis of this disease. Fig. 4 displays an algorithm for the diagnosis of LOPD as per the experts’ recommendations. In patients who are symptomatic and have a known case and mutation within the family, investigation should begin with molecular testing.

Muscle weakness, high serum CK values (up to 15-fold) and a decline in pulmonary function were considered by the experts as criteria for suspicion of LOPD. Consensus was achieved among the experts that a DBS screening test should be conducted in patients with unexplained weakness or suspected LOPD in the MENA region.

It was agreed that one of the following confirmatory tests should be conducted after obtaining a positive DBS screening test or when DBS is inconclusive and there is a high degree of clinical suspicion: enzymatic assay on lymphocytes or fibroblasts, genotyping, or muscle biopsy. GAA enzymatic analysis of fibroblasts was selected as the gold standard, although the logistics of this test were considered a limitation (Table 3). The experts agreed and recommend that local facilities need to be developed in each country to perform the required testing.

**Table 3:** Summary of confirmatory tests for LOPD

Test	Description	Pompe presentation
Lymphocytes for GAA	GAA assayed in purified lymphocyte preparations	GAA activity reduced
Fibroblast cultures for GAA	GAA assayed in cultured fibroblasts from skin biopsy	GAA activity reduced
Genetic testing	Targeted mutational analysis, full sequence analysis, or deletion/duplication analysis	Pathogenic mutations may be detected
Muscle biopsy	Histology or immuno-histology of muscle biopsy samples	Vacuoles that stain positively for glycogen, with accumulation in the lysosomes and cytosol observed in the advanced stages of the disease
		GAA activity reduced
		May be normal or show non-specific changes

*GAA* acid alpha-glucosidase

## Treatment

### Enzyme replacement therapy

ERT with alglucosidase alfa has shown disease stabilization or improvement in patients with LOPD. The Late-Onset Treatment Study (LOTS), which investigated the effect of alglucosidase alfa in 90 LOPD patients, identified improved walking distance and stabilization in pulmonary function in the first 78 weeks of treatment, which was maintained at 104 weeks [23].

In a systematic literature review of LOPD therapy, it was reported that in 368 patients from 21 studies, at least two-thirds were stabilized or had improved CK levels and muscular and/or respiratory function following treatment with alglucosidase alfa [63]. Furthermore, alglucosidase alfa was reported to be well tolerated, with most adverse events categorized as mild to moderate infusion-related reactions. ERT with alglucosidase alfa has been shown to increase life expectancy and survival in IOPD and LOPD [5, 64].

A key challenge in the MENA region is access to treatment and availablilty of alglucosidase alfa.

### Consensus recommendations

#### Initiation

After a diagnosis of Pompe disease by DBS screening and a confirmatory secondary test such as enzyme testing or genetic studies, treatment with alglucosidase alfa should be initiated depending on the status of the patient (Table 4) [6, 7, 65].

**Table 4:** Recommendations for initiation of alglucosidase alfa ERT based on patient status

Patient symptomatology	Recommendations for alglucosidase alfa ERT
Presymptomatic with no signs or symptoms	ERT not necessary. Patient should be monitored every 6 months and ERT initiated if there is evidence of clinical deterioration
Presymptomatic with abnormal muscle imaging or abnormal muscle biopsy	ERT should be considered on a case-by-case basis
Symptomatic with signs or symptoms	ERT should be initiated in patients with muscle weakness or reduced pulmonary function
Patients with markedly advanced disease, who have lost ambulation and are ventilation-dependent	ERT should be administered for 1 year with evaluation of effectiveness

Adapted with permission from Cupler et al. Consensus treatment recommendations for late-onset Pompe disease. Muscle Nerve. 2012;45(3):319–33. [6] © 2011 Wiley Periodicals, Inc

*ERT* enzyme replacement therapy

In presymptomatic patients with no signs or symptoms, ERT is not necessary. However these patients should be monitored every 6 months and treatment with alglucosidase alfa initiated if there is evidence of clinical deterioration in muscle or pulmonary function. Presymptomatic patients who have abnormal muscle imaging or muscle biopsy, and patients without clinical signs but with MRI abnormalities in muscles not traditionally tested (e.g. paraspinal muscles), should be considered for treatment with alglucosidase alfa on a case-by-case basis.

ERT with alglucosidase alfa should be initiated in patients with symptoms or signs of Pompe disease, including early signs of muscle weakness or respiratory insufficiency. ERT should be considered irrespective of whether the patient is using noninvasive ventilation. In advanced patients with severe signs or symptoms, alglucosidase alfa should be administered for 1 year and the effectiveness of the treatment monitored. If patients display a stabilization or improvement in symptoms, ERT should continue. Continuation of ERT in other patients should be considered on a case-by-case basis. It should be noted that there are no randomized controlled trials to display efficacy of ERT in advanced patients, however there are some data in the literature showing some benefit in such patients.

The recommended dosage of alglucosidase alfa is 20 mg/kg body weight administered every 2 weeks as an intravenous infusion [66]. Total infusion volume is determined by patient body weight and should be administered over approximately 4 hrs. Infusion rates should be increased in a step-wise manner, with an initial infusion rate of 1 mg/kg/hr which can be increased by 2 mg/kg/hr every 30 mins until patient tolerance is established, up to a maximum rate of 7 mg/kg/hr.

Anaphylaxis and severe allergic reactions have been observed in patients during, and up to 3 hrs after, alglucosidase alfa infusion. Therefore, it is important that appropriate medical support, including cardiopulmonary resuscitation equipment, is readily available during administration. In the event of anaphylaxis or other severe allergic reactions, immediate discontinuation of administration should be considered and appropriate medical treatment should be initiated. Patients should also be monitored for the development of systemic immune-mediated reactions involving skin and other organs while receiving alglucosidase alfa.

Where it is appropriate and the facilities exist, the goal should be infusions at a local center. Transition of patients to home therapy after 6 months of therapy in an infusion center can be considered based on clinical judgment. This may not be appropriate in patients who have experienced adverse effects due to ERT.

#### Monitoring

Patients with LOPD undergoing ERT with alglucosidase alfa should be clinically monitored every 6 months with the tests described below. Ideally the patients should be assessed by the same examiner at the same time of day (morning or afternoon) to decrease confounding variables. Also the patient should be encouraged to give their best effort. If the patient has experienced a recent intercurrent illness, the assessment should be postponed to allow sufficient recovery to baseline.

##### Manual muscle testing

Manual muscle testing (MMT) should be performed to assess skeletal muscle strength using the Medical Research Council (MRC) grading scale (range 0–5) [67]:

- 0 No contraction
- 1 Flicker or trace of contraction
- 2 Active movement, with gravity eliminated
- 3 Active movement against gravity
- 4- Active movement against gravity and resistance to minimal pressure
- 4 Active movement against gravity and resistance to moderate pressure
- 4+ Active movement against gravity and resistance to strong pressure
- 5 Normal power

Muscle groups that should be assessed include: neck muscles, shoulder abduction, elbow flexion and extension, hip flexion and abduction, knee flexion and extension, and foot dorsal and plantar flexion. For consistency, this should be measured by the same examiner, either a physician or a physiotherapist.

##### Quantitative muscle testing

Where available, quantitative muscle testing (QMT) should be performed using hand-held dynamometry (HHD) [35, 47]. The strength of muscle groups is measured in Newtons and maximum isometric contraction values are assessed with the break technique, in which the examiner applies adequate force to overcome the examinee, thereby producing an eccentric contraction. HHD values obtained for the different muscle groups are expressed as percentages of age- and sex-matched reference values (e.g. [68]).

##### Vital capacity

Around 60 % of LOPD patients have a mild reduction in vital capacity (<80 % predicted) while 30–40 % have a moderate reduction (<60 % predicted) [52]. Screening for diaphragmatic involvement should be performed in the upright and supine positions using a hand-held spirometer. In healthy subjects, vital capacity is 5–10 % less in the supine than the upright position; a drop ≥10 % is suggestive of diaphragmatic weakness and a drop >30 % is suggestive of severe diaphragmatic weakness [53]. If diaphragmatic weakness is found, patients should be referred for formal pulmonary function testing (PFT) and possible sleep studies to assess for nocturnal apnea. Full PFTs should be performed where available including maximum inspiratory pressure (MIP) and maximum expiratory pressure (MEP) as they may be more sensitive to early respiratory muscle weakness [52].

##### Six-minute walk test

The 6-min walk test (6MWT) is used for the objective evaluation of functional exercise capacity; the objective is for the patient to walk as far as possible in 6 mins. Detailed methods are available [69], but briefly, the 6MWT should be performed indoors, along a flat, straight, quiet, corridor 30 m in length, with the length of the corridor marked every 3 m and the turnaround points marked with a cone. The starting line, which marks the beginning and end of each 60-m lap, should be marked on the floor. The patient walks the course back and forth and is permitted to slow down, stop and rest as necessary but encouraged to resume walking as soon as they are able. The healthcare practitioner should time the 6 mins, counting the laps to determine the distance covered, and record patient dyspnea and fatigue using the Borg scale pre- and post-test.

##### Quality of life measurement

The Short Form 36 (SF36) Health Survey has been widely used as a QOL tool in patients with LOPD [70–72], with LOPD patients shown to have a markedly reduced QOL in the physical health domains and a slightly reduced QOL in the mental health domains when compared with the general population [70]. The SF36 Health Survey has been translated and validated in both Arabic [73] and Farsi [74, 75] languages. Ideally, more disease-specific tools (e.g. the IPA/Erasmus MC Pompe survey [37]) would be used, however those have not been standardized or validated in Arabic or Farsi.

##### Laboratory tests

In the first year post-diagnosis, laboratory tests should be performed every 3 months, followed by 6-monthly monitoring in the following years if stable on ERT (Table 5).

**Table 5:** Summary of monitoring assessments to be made during ERT

Test	Frequency
Manual muscle testing	
Quantitative muscle testing	
Vital capacity	Every 6 months
Time on ventilation daily (invasive or noninvasive)	
Six-minute walk test	
Quality of life measurement	
Creatine kinase	Every 3 months in the first year post-diagnosis
Alanine transaminase	
Aspartate transaminase	Every 6 months if stable on ERT
Antibody titers	
Electrocardiography	At baseline and repeated regularly if clinically indicated
Echocardiography	
DEXA (bone mineral density)	
Audiology assessment	

*DEXA* dual-energy X-ray absorptiometry, *ERT* enzyme replacement therapy

##### Creatine kinase

ERT therapy has been shown to stabilize or improve CK levels in LOPD patients and their reduction is considered a useful indicator of response to treatment [63]. However, as CK levels can fluctuate, this may be an unreliable indicator unless a consistent and significant reduction is seen.

##### GAA antibody titers

A number of factors have been identified as the cause of poor response to ERT, one of which is high, sustained antibody titers (HSAT) [72]. However, while HSAT has been shown to correlate with poor response to ERT in IOPD [76, 77], knowledge of the role of antibodies in the LOPD population is limited. A recent study has shown that HSAT in a subset of LOPD patients is associated with clinical decline; however further studies are required to fully understand impact of antibodies in LOPD [72].

##### Audiology assessment

While considered rare, there are reports of mild hearing loss in LOPD patients [45, 46]. It is therefore recommended that an audiology assessment is performed at baseline and then as clinically indicated.

### Algorithm for treatment of LOPD

**Fig. 5:**
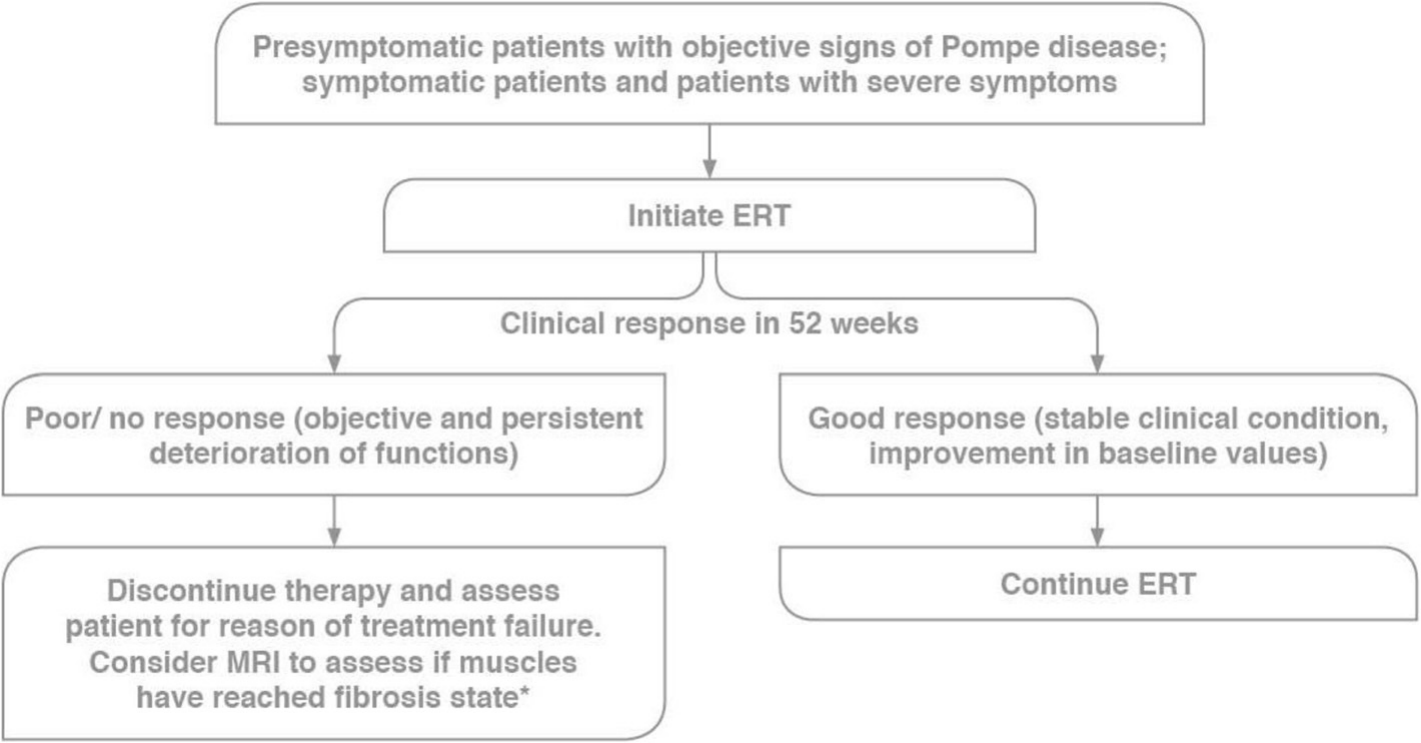
Algorithm for treatment of LOPD. Abbreviations: ERT, enzyme replacement therapy; MRI, magnetic resonance imaging. *Restarting of ERT with alglucosidase alfa should be considered if there is rapid deterioration post-discontinuation

In asymptomatic patients with a confirmed diagnosis of Pompe disease, the experts recommended conducting PFTs and muscle strength tests; close monitoring for objective signs and symptoms every 6 months was also recommended (Table 5). Upon onset of signs or symptoms, the experts recommended commencement of ERT with alglucosidase alfa (Fig. 5).

#### Treatment failure

The experts defined treatment failure as objective and persistent deterioration of functions following 52 weeks of therapy. Evaluation should include physical examination, assessment for alternative etiologies of disease progression (e.g. hypothyroidism, vitamin D deficiency), laboratory studies, muscle MRI, GAA antibody titers, and possible muscle biopsy. In non-responders, treatment should be discontinued and progress monitored if an alternative explanation is not determined. Monitoring should be continued after ERT discontinuation. Restarting ERT should be considered if the rate of deterioration increases after discontinuation of ERT. Individualized therapeutic decisions such as high-dose ERT are at the discretion of the treating physician.

## Management of components of LOPD

### Respiratory

#### Sleep study

A sleep study should be performed when there is an abnormal drop of >10 % in FVC between the upright and supine position, or when there are symptoms suggestive of nocturnal hypoventilation or sleep apnea. If the sleep study demonstrates abnormalities then a pulmonologist with interest in neuromuscular disease should assess the patient for possible bi-level positive airway pressure (BPAP) or other noninvasive ventilator support to improve nocturnal respiration.

### Cardiac

#### Electrocardiography

A study of the clinical spectrum of LOPD patients identified three subjects who suffered from arrhythmias due to Wolff–Parkinson–White syndrome despite no echocardiographic signs of cardiomyopathy [1] and this should therefore be considered during cardiac monitoring of LOPD patients. Some patients may have short PR interval in their electrocardiogram (ECG) secondary to enhanced conduction [42]. Others may have abnormalities reflecting their underlying cardiac disease (e.g. bundle branch block), rather than Pompe disease. As such, ECG should be performed at baseline, and repeated regularly only if clinically indicated.

#### Echocardiography

Unlike patients with the infantile form, patients with LOPD do not typically develop hypertrophy of the left ventricle (LVH) [37, 42, 78]. In a study of 75 adult patients with hypertrophic cardiomyopathy, none had genetic evidence of Pompe disease [78]. Neither Hagemans et al. nor Soliman et al. found LVH in their series of adult patients with LOPD [37, 42]. Most of the cardiac abnormalities described in patients with LOPD are explained by concurrent underlying diseases, rather than LOPD [42]. ECGs should be performed at baseline, then as clinically indicated.

### Gastrointestinal issues

#### Gastrointestinal motility dysfunction

A 2010 study reported gastrointestinal (GI) symptoms of chronic constipation and GI reflux in four out of six LOPD patients studied at a single institution [44]. At 1 year follow-up, the patients reported a marked improvement of intestinal symptoms. Another study reported three LOPD patients with chronic diarrhea, postprandial bloating, and abdominal pain [79]. Two of the patients reported fecal incontinence while the other reported synchronous vomiting and diarrhea; however all GI symptoms were reported to be resolved within the first 6 months of ERT.

#### Swallowing dysfunction

A retrospective review of LOPD patients evaluated at the neuromuscular clinic at Duke University Medical Center identified 12 patients, three of whom had symptoms of oropharyngeal dysphagia [41]. This was categorized as mild in two cases and severe in one, with the degree of swallowing impairment appearing to correlate with overall physical strength and function. Moderate or severe lingual weakness was found to be associated with dysphagia, and swallowing difficulties were generally more oral than pharyngeal in nature. It was concluded that screening for symptoms of dysphagia could help reduce morbidity and mortality, while improving understanding of the LOPD phenotype. A recent study has further demonstrated evidence of lingual weakness in LOPD, with a quantitative assessment showing lingual weakness in 80 % of the sample [80].

### Diet/Nutrition

At baseline, a study of 17 patients with LOPD undergoing ERT identified increased fat mass in five patients in severe disease stage despite normal body mass index (BMI) [81]. Fat mass was found to correlate inversely, and lean mass directly, with CK, prealbumin, and albumin levels. ERT resulted in significant increases in BMI and fat mass, and a trend towards increased lean mass. Prealbumin and albumin levels increased as early as after the first months of ERT. This study highlighted that BMI may underestimate fat mass in patients in severe stage of disease, due to altered body composition. Furthermore, patients may have a relative protein malnutrition which is reversed by ERT, this reflecting restoration of normal muscle metabolic pathways. The increased BMI may indicate a reduction in energy consumption during exercise or respiration, along with clinical improvement.

#### High protein diet

A high-protein diet has been shown to have beneficial effects in Pompe patients [82]. A ventilator-dependent Pompe patient fed a general diet supplemented with branched-chain amino acids, which are the principal amino acids involved in muscle protein synthesis and utilization, showed improved respiratory function and muscle strength, resulting in daytime weaning form the ventilator. The potential advantages of a branched-chain amino acids-supplemented liquid diet over a high-protein diet include theoretical sparing of amino acids required for muscle protein synthesis by providing higher concentrations of postprandial branched-chain amino acids in the circulation, and better tolerance by a ventilator-dependent or debilitated patient.

#### Supplements

Supplementation of LOPD patients with L-alanine reduces protein turnover and catabolism [83]. A study of five subjects found that L-alanine supplementation decreased resting energy expenditure, leucine flux, and leucine oxidation to levels lower than those observed in control subjects. L-alanine has not been studied in combination with ERT.

#### Gastrostomy tube

In patients where muscular weakness affects the ability to eat or where breathing problems interfere with eating, a gastrostomy tube (G-tube) may be necessary for providing adequate nutrition [84]. These may also be used to improve nutrition in some patients who have normal swallowing function.

### Exercise

An uncontrolled prospective study of 34 patients found that compliance to high-protein and low-carbohydrate nutrition and exercise therapy significantly slowed deterioration of muscle function as measured by the Walton scale, and improved the natural history of disease progression [85]. Another study investigated the effect of exercise training on muscular strength and body composition in five patients with LOPD receiving ERT who underwent a 20-week program of supervised aerobic and progressive resistance exercise training. Exercise training resulted in a significant increase in muscular strength and 6-min walking distance despite no change in total and lower extremity lean body mass, highlighting the benefits of exercise training in patients with LOPD on ERT [86].

Studies have also sought to investigate whether exercise training during ERT infusion increases effectiveness of therapy, however in a study of five LOPD patients following a 6-month period of exercise training, no significant functional changes in body composition, isometric strength, or 6-min walking distance were identified [87].

### Bone

Low bone mineral density and osteoporosis have been reported in LOPD patients [47]. Studies of the effect of ERT on bone mineral density suggest that ERT administration may moderately improve bone mineral density in some, but not all, LOPD patients [88, 89].

#### DEXA scan

Due to this bone mineral density component of LOPD, it is recommended that patients are screened at baseline with dual-energy X-ray absorptiometry (DEXA) and then as clinically indicated [6, 47, 52].

#### Vitamin D plus calcium +/− bisphosphonates

Patients with abnormal DEXA z-scores should be supplemented with vitamin D and calcium [47]. There is insufficient evidence to recommend bisphosphonates as a preventative therapy, however their use in LOPD patients should follow the same guidelines as for the general population [6, 52].

### Others

#### Possible increase in cerebral vascular anomalies

An investigation into cerebrovascular anomalies in six LOPD patients identified brain vascular anomalies, including dolichoectasia of the basilar artery and ectasia of internal carotids, in four out of the six patients [44]. Two patients had clinical signs related to the arteriopathy, including partial paralysis of the third cranial nerve and transient ischemic attacks. ERT at 1 year follow-up was found to have no effect on the size of cerebral vessels and the study concluded that as intracranial artery abnormalities are not infrequent in patients with LOPD, they should be specifically investigated in the presence of unexplained CNS symptoms.

A 2008 case report characterized the thrombotic complications of a basilar artery aneurysm in a young adult with Pompe disease, with the authors concluding that screening of intra-cerebral vessels to potentially diagnose thrombotic and thromboembolic complications may be considered in young adults with Pompe disease [90].

A study published in 2011 reported five LOPD patients with aortic arteriopathy involving primarily the ascending thoracic aorta, highlighting that aneurysmal dilatation of the thoracic aorta is an underreported vascular complication of LOPD [91]. The authors suggested that chest X-ray and echocardiography should be incorporated as initial screening tools for aortic aneurysms in patients with LOPD. Furthermore, contrast-mediated thoracic CT or MRI may be necessary when ectasia is suspected or the ascending aorta is not visualized.

#### Genetic counseling

As Pompe is an inherited autosomal recessive disease, it is important to undertake genetic counseling with families that are known to be carriers or when there is diagnosis of a new case in a family. The most common inheritance scenario in Pompe disease is where both parents are asymptomatic carriers; in this instance the risk of Pompe disease is 1 in 4 (25 %), the risk that the child will become a carrier is 2 in 4 (50 %), and the risk that the child will be unaffected is 1 in 4 (25 %). As Pompe disease is an autosomal recessive disorder, typically counseling can be performed with an emphasis on prenatal testing and early diagnosis in the newborn.

### Pregnancy

Pregnancies in Pompe disease patients, including cases of spinal anesthesia and Cesarean section, have been shown to result in the birth of healthy infants [92], however the pregnancy should be considered high risk. In 2008 it was reported that a 31-year-old patient with known Pompe disease with symptoms and signs of respiratory dysfunction as well as preeclampsia underwent urgent Cesarean section under regional anesthesia, resulting in the birth of a healthy baby girl.

There are limited data on the use of alglucosidase alfa in pregnant women; however studies in animals have shown reproductive toxicity [93]. While safe continuation of ERT during pregnancy and lactation has been reported in a case study [94], the potential risk for humans is unknown and the decision to use alglucosidase alfa during pregnancy needs to be made on an individual basis [93].

A case study reported a primiparous 40-year-old woman diagnosed with Pompe disease whose clinical condition remained fairly stable until the 25th gestational week, after which problems with mobility and respiration were experienced. Fetal growth, as monitored by ultrasound, was reported to be normal and a healthy boy was born at a gestational age of 37 weeks and 5 days by elective Cesarean section. There were no maternal complications and the child developed normally. The mother’s physical condition at 1 year post-partum was similar to prior to her pregnancy. Pharmacokinetic studies following enzyme infusion showed that alglucosidase alfa was secreted into the breast milk, with enzyme activity in the breast milk returning to the pre-infusion level after 24 hrs.

Alglucosidase alfa may be excreted in breast milk and as there are limited data on the effects in neonates exposed to alglucosidase alfa via breast milk, cessation of breastfeeding during use of alglucosidase alfa should be advised [93].

## Future therapies for LOPD

### NeoGAA

NeoGAA has been designed to improve delivery of rhGAA to lysosomes by remodeling the carbohydrate moieties on the enzyme to exhibit a high-affinity ligand for the cation-independent mannose 6-phosphate receptor which is responsible for cellular uptake [95]. *In vivo* studies of administration to Pompe mice demonstrate improved clearance of glycogen from affected muscles when compared with the unmodified rhGAA, suggesting promise in enhancing the efficacy of ERT for Pompe disease [95]. NeoGAA is currently undergoing Phase I clinical trials in humans to assess tolerability, pharmacokinetics, and pharmacodynamics [96].

### GAA fusion protein

BMN 701 is a fusion protein of insulin-like growth factor and GAA which has a high affinity for the cation-independent mannose 6-phosphate receptor [97]. BMN 701 has biochemical properties similar to rhGAA but is delivered more effectively to the lysosomes. *In vivo* studies have shown improved clearance of glycogen in Pompe mouse models and the therapy is now in Phase III trials [97, 98].

### Chaperone therapy

Chaperone therapy, also known as enzyme enhancement therapy (EET), is where pharmacological protein chaperones are co-administered with a protein therapy to improve efficacy. *In vitro* and *in vivo* studies show that co-administration of rhGAA and chaperones such as N-butyldeoxynojirimycin (NB-DNJ) [99] or AT2220 (duvoglustat HCl) [100] results in more efficient correction of enzyme activity, with improved delivery to lysosomes, enhanced enzyme maturation, increased enzyme stability, and greater glycogen reduction. A recently reported Phase II trial showed that co-administration of duvoglustat HCl and rhGAA to Pompe patients increased active plasma rhGAA AUC levels in all Pompe patients and that approximately 70 % of patients had increased muscle rhGAA activity [101].

Chaperone therapy can also be given alone, with the idea that the chaperone will bind to the partially active molecule and traffic it to the lysosome where the acidic pH will dislodge the chaperone and permit the defective enzyme to work [102]. Normally, defective enzymes are tagged for destruction, however the chaperone prevents their degradation. While Phase II trials of chaperone monotherapy have been initiated, they were subsequently discontinued [103] due to safety concerns and the future of chaperone monotherapy remains unclear.

### Gene therapy

Adeno-associated virus (AAV)-mediated delivery of GAA has been shown to result in partial biochemical correction of the skeletal muscles and diaphragm and improved motor function in animal models of Pompe disease [104, 105].

Results from a Phase I/II study of AAV-GAA in a severely affected patient group of five children dependent on mechanical ventilation despite ERT suggest gene therapy may be safe and may lead to modest improvements in volitional ventilatory performance measures [106]. Gene therapy led to significant increases in unassisted tidal volume, as well as an increase in patient tolerance for duration of unassisted breathing. However, further studies in larger, more diverse patient populations are required to understand the efficacy and safety of gene therapy in Pompe disease.

## Discussion

This consensus manuscript is the first to provide region-specific guidelines for the MENA region on the diagnosis and treatment of LOPD. They take into account the challenges that are unique to the region; namely delayed diagnosis, access to diagnostic tools, and access to treatment. Considering that the prevalence of LOPD in the region is at least as high as in other countries, and is likely to be higher, the publication of regional guidelines serves to facilitate the prompt diagnosis, appropriate treatment, and proper monitoring that will improve survival and patient outcomes.

There is a lack of awareness of LOPD in the region, which potentially leads to missed cases, delayed diagnosis, and delayed treatment. There is therefore a need to improve the knowledge of physicians and adult neurologists so that they are aware of LOPD as a potential differential diagnosis in patients presenting with limb–girdle disease.

Another challenge faced within the region is the lack of patient education. The authors are aware that there is a reluctance of patients to seek medical attention in the early stages of the disease, which leads to delayed diagnosis and treatment and poor patient outcomes. This is potentially associated with disease stigma and denial of the disease, which also result in a lack of discussion within families and a missed opportunity for family screening. Another patient challenge in the region is the refusal of lifelong treatment or non-compliance with physician recommendations, especially if the disease is mild in severity. Patients need to be educated to be aware of the disease and the need to seek prompt medical attention.

The authors hope that dissemination of these guidelines will assist healthcare professionals in achieving prompt diagnosis, appropriate treatment, and proper follow-up of LOPD patients in order to reduce the burden of the disease.

## Abbreviations

6MWT: 6-minute walk test
AAV: Adeno-associated virus
ALT: Alanine aminotransferase
AMD: Acid maltase deficiency
AST: Asparate aminotransferase
BMI: Body mass index
BPAP: Bi-level positive airway pressure
CHO: Chinese hamster ovary
CK: Creatine kinase
DBS: Dried blood spot
DEXA: Dual-energy X-ray absorptiometry
ECG: Electrocardiogram
EET: Enzyme enhancement therapy
EMG: Electromyograph
ERT: Enzyme replacement therapy
FVC: Forced vital capacity
GAA: Acid alpha-glucosidase
GI: Gastrointestinal
GSDII: Glycogen storage disease type II
HHD: Hand-held dynamometry
HSAT: High, sustained antibody titer
IOPD: Infantile-onset Pompe disease
LDH: Lactate dehydrogenase
LOPD: Late-onset Pompe disease
LOTS: Late-Onset Treatment Study
LVH: Left ventricular function
MENA: Middle East and North Africa
MGA: Maltase gluco-amylase
MMT: Manual muscle testing
MRC: Medical Research Council
MRI: Magnetic resonance imaging
PFT: Pulmonary function test
QMT: Quantitative muscle testing
QOL: Quality of life
rhGAA: Recombinant human GAA
RSS: Rigid spine syndrome
SF36: Short Form 36.
